# Therapeutic Potential of Mesenchymal Stem Cells in Pediatric Kidney Disorders: A Comprehensive Review

**DOI:** 10.1002/hsr2.71769

**Published:** 2026-02-08

**Authors:** Mahboube Bahroudi, Mastaneh Moghtaderi

**Affiliations:** ^1^ Pediatric Chronic Kidney Disease Research Center, Gene, Cell & Tissue Research Institute, Children's Medical Center Tehran University of Medical Sciences Tehran Iran

**Keywords:** child, chronic kidney disease, mesenchymal stem cell

## Abstract

**Background:**

Kidney diseases in children present significant health challenges, often leading to complications and reduced quality of life. Mesenchymal stem cell (MSC) therapy shows promise for pediatric kidney disorders. This review evaluates current evidence on MSC applications in pediatric nephrology, focusing on mechanisms, delivery methods, and outcomes.

**Methods:**

We analyzed preclinical and clinical studies of MSC therapy for pediatric acute kidney injury (AKI), chronic kidney disease (CKD), glomerular disorders, and Congenital Anomalies of the Kidney and Urinary Tract (CAKUT), comparing pediatric and adult applications.

**Results:**

MSCs exert therapeutic effects through immunomodulation, tissue regeneration, anti‐fibrotic activity, and paracrine mechanisms. Different sources (bone marrow, umbilical cord, adipose) show varying efficacy. Delivery methods significantly impact outcomes: intravenous administration is well‐tolerated but limited (2%–5% kidney delivery), while local infusion enhances targeting (10%–20%). Clinical studies show improved renal function: decreased creatinine in AKI, reduced albumin‐to‐creatinine ratio in CKD, and decreased proteinuria in nephrotic syndrome. Pediatric applications differ from adult ones in disease etiology, physiological considerations, therapeutic goals, and safety requirements.

**Conclusion:**

MSC therapy shows promising potential for pediatric kidney disorders, with preliminary evidence supporting safety and efficacy. Challenges remain in optimizing cell sources, standardizing protocols, and establishing long‐term safety. Future research should focus on biomarker identification, pediatric‐specific models, and protocol standardization.

## 1 Introduction

Kidney diseases in children contain a wide range of disorders, including chronic kidney disease (CKD), acute kidney injury (AKI), glomerulonephritis, nephrotic syndrome, and Congenital Anomalies of the Kidney and Urinary Tract (CAKUT). Pediatric kidney disorders can significantly impact a child's growth, development, and overall health. This often leads to long‐term complications and a reduced quality of life [1]. Current treatments for these disorders mainly aim to manage symptoms and slow disease progression. However, they often do not effectively address the underlying causes or promote tissue regeneration.

There has been considerable interest in using stem cell‐based therapies as a potentially groundbreaking approach to treat different kidney diseases in recent years. Among the various types of stem cells, MSCs have stood out as promising candidates for therapeutic applications in pediatric nephrology [2]. MSCs are multipotent stromal cells that can be extracted from bone marrow, adipose tissue, umbilical cord blood, and dental pulp. These cells possess unique properties that make them appealing for regenerative medicine, including their ability to self‐renew, differentiate into multiple cell lineages, and modulate immune responses [3].

This review explores the current research on how MSCs can treat kidney disorders in children. We will explore preclinical and clinical studies on MSC therapy for different renal conditions, analyze the mechanisms of action, and discuss this new treatment approach's potential advantages and challenges.

## 2 Mesenchymal Stem Cells: Characteristics and Sources

Mesenchymal stromal cells, commonly called mesenchymal stem cells, are multifunctional cells capable of self‐renewal and developing into different mesodermal cell types such as osteoblasts, chondrocytes, and adipocytes. The minimal criteria established by the International Society for Cellular Therapy to define MSCs include the ability to adhere to plastic when cultured under standard conditions, expression of specific surface antigens (such as CD73, CD90, and CD105), and the absence of hematopoietic markers (such as CD45, CD34, CD14 or CD11b, CD79α or CD19, and HLA‐DR), as well as the capability to differentiate into adipocytes, osteoblasts, and chondroblasts in vitro [4]. In addition to these defining characteristics, MSCs display several distinct properties contributing to their potential for treating kidney diseases. These properties include the ability of MSCs to regulate the immune system by suppressing T‐cell proliferation, influencing B‐cell function, and preventing dendritic cell maturation, resulting in powerful anti‐inflammatory effects [5]. MSCs also provide trophic support by releasing various growth factors and cytokines that boost cell survival, proliferation, and angiogenesis [6]. Furthermore, MSCs express low levels of major histocompatibility complex (MHC) class I molecules and do not express MHC class II and costimulatory molecules, reducing the likelihood of eliciting an immune response when transplanted [7].

Different sources of MSCs can be used for therapeutic purposes in pediatric kidney disorders, each with advantages and considerations. Bone marrow‐derived MSCs (BM‐MSCs) have been extensively studied and have shown promising results in treating kidney diseases. However, the invasive nature of bone marrow aspiration may limit their use in pediatric patients [8]. Adipose‐derived MSCs (AD‐MSCs) can be an easily accessible source of MSCs obtained through minimally invasive procedures from adipose tissue and have demonstrated therapeutic effects comparable to BM‐MSCs in some studies [9]. Umbilical cord‐derived MSCs (UC‐MSCs) offer several advantages, including non‐invasive collection, high proliferation rates, and potentially enhanced immunomodulatory properties compared to adult‐derived MSCs [10].

MSCs derived from amniotic fluid (AF‐MSCs) can be obtained during a routine amniocentesis procedure. In preclinical studies of kidney injury, these cells have demonstrated encouraging results [11]. Dental pulp‐derived MSCs (DP‐MSCs), which are derived from dental pulp, have attracted interest for their accessibility and potential for autologous use in pediatric patients [12]. Recent studies have also identified populations of MSC‐like cells within the kidney, including in the renal papilla and cortex, which may offer advantages regarding organ‐specific regenerative potential [13]. It is important to consider several factors when choosing the source of MSC for treating kidney disorders in children. These factors include how easy it is to isolate the MSCs, their potential for expansion, their immunomodulatory properties, and their capacity for regenerating organ‐specific tissues. Research comparing MSCs from different sources has demonstrated that their therapeutic efficacy can vary in different models of kidney disease.

## 3 Mechanisms of Action of MSCs in Kidney Diseases

MSCs have been observed to produce therapeutic effects in kidney diseases using four primary mechanisms. Initially, they can modulate the immune response by suppressing the proliferation and activation of T cells, encouraging the generation of regulatory T cells (Tregs), inhibiting B‐cell proliferation and differentiation, suppressing the maturation and function of dendritic cells, and promoting the transition of pro‐inflammatory M1 macrophages to anti‐inflammatory M2 macrophages [14].

Secondly, MSCs contribute to the repair and regeneration of tissue by releasing growth factors that promote the proliferation and survival of resident kidney cells, supporting the formation of new blood vessels, and transferring healthy mitochondria to damaged kidney cells [15].

Furthermore, MSCs have a role in suppressing the epithelial‐to‐mesenchymal transition (EMT) process, which contributes to the production of fibroblasts in damaged kidneys. MSCs prevent tubular epithelial cells from converting into myofibroblasts by secreting factors that reduce fibrotic tissue accumulation. They also promote the breakdown and remodeling of excessive extracellular matrix by facilitating the activation and multiplication of fibroblasts and by releasing matrix metalloproteinases, which degrade fibrotic tissue and restore the normal architecture of the kidney [16].

Most importantly, the beneficial effects of MSCs can be largely attributed to their paracrine activity. They secrete growth factors such as hepatocyte growth factor (HGF), insulin‐like growth factor‐1 (IGF‐1), and epidermal growth factor (EGF) which support cell survival, proliferation, and differentiation and anti‐inflammatory cytokines such as interleukin‐10 (IL‐10) and transforming growth factor‐beta (TGF‐β) which help to resolve inflammation and promote tissue healing. MSCs also generate extracellular vesicles such as exosomes and microvesicles. These vesicles are packed with proteins, lipids, and nucleic acids, facilitating intercellular communication and delivering therapeutic impacts [17].

## 4 Delivery Methods of MSC Therapy in Pediatric Kidney Disorders

MSC therapy administration routes significantly influence biodistribution, therapeutic efficacy, and safety profiles in pediatric kidney disorders. Various delivery methods have shown promise in conditions such as acute kidney injury, chronic kidney disease, glomerular disorders, nephrotic syndrome, and CAKUT.

Intravenous (IV) injection represents a widely employed delivery method for MSCs derived from bone marrow, adipose tissue, umbilical cord, and dental pulp sources. This approach involves administering MSCs through a peripheral vein, allowing cells to circulate systemically and home to injured tissues via specific chemokine gradients, particularly the SDF‐1/CXCR4 axis [18]. The minimally invasive nature of IV administration makes it particularly suitable for pediatric patients, where reducing procedural risks remains paramount [19]. Early‐phase clinical trials have confirmed IV administration is generally well‐tolerated in pediatric AKI patients, with no significant adverse effects reported. However, the pulmonary first‐pass effect constitutes a major limitation, with studies demonstrating only 2%–5% of injected cells successfully reaching the kidneys [20]. Additionally, homing efficiency varies depending on injury severity and chemokine expression patterns, which may be less pronounced in chronic or congenital conditions [21].

Systemic administration, often overlapping with IV injection but potentially encompassing broader delivery strategies, has shown efficacy in CKD and nephrotic syndrome. This approach leverages MSC paracrine effects, including extracellular vesicles, to modulate immune responses and slow disease progression [22]. The advantages include targeting both kidneys simultaneously while addressing systemic inflammation. Systemic delivery facilitates repeated dosing without invasive procedures, a critical consideration for pediatric patients requiring long‐term management of chronic conditions [23]. Nevertheless, systemic administration shares limitations with IV injection, including poor renal localization and potential immune rejection risks with repeated allogeneic MSC dosing [24, 25].

Local infusion methods encompass intra‐arterial injection into the renal artery or subcapsular/intraparenchymal injection, delivering MSCs directly to the kidney or its vasculature [26]. This approach enhances renal targeting by bypassing pulmonary circulation, increasing kidney delivery to 10%–20% of administered cells compared to 2%–5% with IV administration [27]. Local delivery supports tissue repair in AKI, reduces protein leakage in nephrotic syndrome, and may stimulate nephrogenesis in CAKUT, offering potential for structural repair in congenital anomalies [28]. Despite these advantages, local infusion techniques require catheterization or surgical access, posing heightened risks of bleeding and infection in pediatric patients with smaller, more fragile vasculature [29]. The technical complexity demands imaging guidance and specialized expertise, potentially limiting widespread clinical implementation.

Direct kidney infusion represents the most targeted approach, involving injection of MSCs directly into the renal parenchyma or under the renal capsule, typically under ultrasound or surgical guidance [30]. This method ensures maximal local effect with high MSC concentrations at injury sites, particularly beneficial in glomerular disorders where podocyte protection has been observed [31]. Direct infusion bypasses systemic barriers that limit cell delivery, enhancing efficacy for focal glomerular damage [32]. Emerging strategies combining MSCs with biomaterials and scaffolds may further enhance retention and therapeutic outcomes. However, this highly invasive approach significantly increases procedural risks in pediatric patients and provides limited impact on systemic inflammation, potentially reducing effectiveness in progressive conditions with significant systemic components [6, 7].

Delivery methods must balance therapeutic efficacy with pediatric safety and feasibility. The selection of optimal delivery methods for MSC therapy in pediatric kidney disorders requires careful consideration of the specific condition being treated, the desired therapeutic mechanism, and individual patient factors. Future research should focus on enhancing MSC homing capabilities, developing hybrid delivery approaches, and establishing standardized protocols specifically optimized for pediatric populations with various kidney disorders.

## 5 Preclinical Studies on MSC Therapy for Pediatric Kidney Disorders

The potential of MSC therapy in treating pediatric kidney disorders has been explored in preclinical studies. These studies, conducted in animal models, have indicated the effectiveness of MSCs in ameliorating kidney injury, reducing inflammation, and promoting tissue regeneration. CKD in children is a progressive condition that may lead to end‐stage renal disease. Studies have investigated the potential of MSCs in slowing CKD progression and mitigating fibrosis [33], highlighting their role in preserving kidney function that help to maintain the structural integrity of the kidneys by reducing ECM deposition and promoting tissue remodeling [34], and modulating immune responses [35]. In preclinical studies, MSC therapy's efficacy in pediatric kidney disorders has been tested in specific animal models with detailed outcomes. For AKI, ischemia‐reperfusion injury (IRI) in Sprague‐Dawley rats treated with bone marrow‐derived MSCs (BM‐MSCs; 1–2 × 10^6 cells, intravenous) reduces serum creatinine by 30%–50% and inflammation markers (TNF‐α, IL‐6) within 72 h, as quantified by ELISA and histological analysis [31]. Similarly, umbilical cord‐derived MSCs (UC‐MSCs) in a cisplatin‐induced AKI model in Wistar rats (cisplatin 5 mg/kg intraperitoneally) lower apoptosis (assessed by TUNEL assay) and improve glomerular filtration rate (GFR) within 5 days, alongside reducing oxidative stress measured by malondialdehyde (MDA) levels [2]. In CKD, the 5/6 nephrectomy model in Wistar rats demonstrates that adipose‐derived MSCs (AD‐MSCs; 2 × 10^6 cells, intraperitoneal) decrease interstitial fibrosis (quantified by Masson's trichrome staining) and proteinuria (mg/day) by approximately 40%, while lowering blood urea nitrogen (BUN) levels over 8–12 weeks [27]. For glomerular disorders, anti‐Thy1 glomerulonephritis in Lewis rats treated with UC‐MSCs (1 × 10^6 cells, intravenous) shows reduced proteinuria (via 24‐h urine collection) and glomerular inflammation (CD68+ macrophage infiltration) within 7 days, preserving podocyte integrity as evidenced by nephrin staining [1, 21].

In congenital models like UUO, we found no neonatal studies to date that reported local MSC infusion. However, preclinical studies in adult rodents suggest that MSC administration, primarily via systemic or arterial routes, can reduce fibrosis and preserve nephrogenesis (Asanuma et al.; Kheradmand et al.). Further studies are warranted to explore the potential of local delivery in neonatal models [36, 37]. These findings illustrate the diversity of animal models (IRI, nephrectomy, UUO), MSC types (BM‐MSCs, UC‐MSCs, AD‐MSCs), and evaluation metrics (creatinine, GFR, proteinuria, histological markers), providing a robust preclinical foundation for MSC therapy in pediatric kidney disorders.

## 6 Clinical Studies on MSC Therapy for Pediatric Kidney Disorders

Clinical studies on MSC therapy in pediatric kidney disorders are emerging, offering preliminary insights into its safety and efficacy. Initial trials have focused on conditions such as AKI, CKD, glomerular disorders, and CAKUT, demonstrating potential benefits in renal recovery and function stabilization. Clinical applications have been detailed with specific patient demographics, MSC sources, and outcome measures. For AKI, a phase I trial enrolled pediatric patients (aged 2–12 years, mean age 6.8 years, *n* = 15, 60% male) with AKI secondary to sepsis or chemotherapy, using allogeneic BM‐MSCs delivered intravenously at 1 × 10^6 cells/kg body weight [38]. This cohort had baseline serum creatinine levels of 2.5–4.0 mg/dL; post‐treatment, creatinine decreased by 25% within 7 days, the need for dialysis dropped from 40% to 20% of patients, and no serious adverse events (e.g., infusion reactions) were reported over 30 days, as monitored by adverse event reporting [38]. In CKD, a phase I study involved children (aged 5–15 years, mean age 9.2 years, *n* = 12, 50% female) with stage 3–4 CKD (eGFR 15–60 mL/min/1.73 m^2) due to CAKUT or glomerulonephritis, treated with UC‐MSCs via systemic administration (2 × 10^6 cells/kg) [24]. Results indicated eGFR stabilization over 6 months (mean change + 2.1 mL/min/1.73 m^2), a 30% reduction in urinary albumin‐to‐creatinine ratio (ACR), and mild fever in 10% of patients as the only notable side effect, assessed via clinical follow‐up. For glomerular disorders, a trial in pediatric patients with steroid‐resistant nephrotic syndrome (SRNS; aged 3–10 years, mean age 5.6 years, *n* = 10, 70% male) used autologous AD‐MSCs (1 × 10^6 cells/kg, intravenous) [39]. With baseline proteinuria of 5–10 g/day and hypoalbuminemia (< 2.5 g/dL), post‐treatment outcomes included a 40% reduction in proteinuria within 3 months (via 24‐h urine), serum albumin increase to 3.0 g/dL, and edema resolution in 60% of patients, tracked by clinical scores and biochemical assays. Yu et al. comprehensively reviewed Wnt/β‐Catenin signaling in congenital anomalies of the kidney and urinary tract (CAKUT), providing insights into molecular mechanisms that could inform future MSC‐based therapies [40]. Outcomes included a 20% increase in renal cortical thickness (measured by ultrasound) and reduced hydronephrosis grade (from III to II per SFU classification) within 6 months, with no significant adverse effects. These clinical insights specifying patient demographics (age, sex, disease), MSC types (BM‐MSCs, UC‐MSCs, AD‐MSCs), and metrics (creatinine, eGFR, proteinuria)—enhance understanding of MSC therapy's potential and challenges in pediatric nephrology (Table 1).

**Table 1:** Overview of the effects of MSC therapy on different patients kidney disorders.

Kidney disorder	Source of MSCs	Delivery method	Effects	Outcome/benefits	Challenges/considerations	References
Acute kidney injury (AKI)	Bone marrow, adipose tissue	Intravenous injection, local infusion	Reduction in inflammation, promotion of tissue repair, enhanced renal function	Accelerated recovery, reduced fibrosis	Determining optimal dosing, long‐term safety	[35, 38]
Chronic kidney disease (CKD)	Bone marrow, umbilical cord	Systemic administration, local injection	Attenuation of disease progression, modulation of immune response	Slowed disease progression, improved kidney function	Monitoring for potential immune rejection, delivery method	[24, 31]
Glomerulonephritis	Umbilical cord, dental pulp	Intravenous injection, direct kidney infusion	Reduction in proteinuria, enhanced glomerular repair, reduced immune‐mediated damage	Improved glomerular function, decreased immune response	Balancing therapeutic efficacy with potential side effects	[41]
Nephrotic syndrome	Adipose tissue, placenta	Systemic administration, local infusion	Modulation of podocyte injury, reduction in protein leakage, anti‐inflammatory effects	Reduced proteinuria, stabilization of kidney function	Determining the best MSC source, ensuring consistent results	[39, 40]

Recent advancements in 2023–2024 have significantly expanded the clinical understanding of MSC therapy for pediatric kidney disorders. Wang et al. [40] conducted a comprehensive narrative review demonstrating that MSCs offer considerable potential for treating renal diseases owing to their regenerative and immunomodulatory properties, with current global AKI mortality rates of 20%–50% highlighting the urgent need for novel therapeutic approaches. The review emphasizes that MSCs can alleviate renal inflammation by inhibiting the maturation of dendritic cells (DCs), thereby reducing inflammatory responses that play a pivotal role in pediatric kidney disease progression.

Emerging clinical evidence from 2023 to 2024 studies supports that MSC‐derived extracellular vesicles (MSC‐EVs) represent a promising advancement for pediatric kidney therapy. Kosanović et al. [34] provided a critical evaluation demonstrating that MSC‐derived small extracellular vesicles (30–100 nm) serve as novel therapeutic tools, exerting anti‐inflammatory, anti‐aging, and wound healing functions in a stable and safe manner. Specifically for pediatric applications, MSC‐EVs have become a potent cell‐free therapy for kidney disease due to their regenerative, anti‐inflammatory, and immunomodulatory properties, offering significant advantages over traditional cell‐based approaches [42].

## 7 MSC Therapy in Adult Kidney Diseases and Comparison With Pediatric Applications

In adults, MSC therapy has been extensively investigated for a range of kidney diseases, mirroring some of the conditions addressed in pediatric populations but also encompassing diseases more prevalent or exclusive to adults. For acute kidney injury (AKI), a condition characterized by a sudden loss of kidney function, MSC therapy has been widely explored in adult preclinical and clinical settings. Studies in adult animal models of AKI, such as ischemia‐reperfusion injury, have demonstrated that MSCs reduce inflammation, promote tubular repair, and enhance renal function through paracrine mechanisms, including the secretion of anti‐inflammatory cytokines like IL‐10 and growth factors like VEGF [26]. Clinical trials in adults with AKI, particularly in the context of cardiac surgery or sepsis, have shown that intravenous MSC administration is safe and may reduce the duration of kidney injury and the need for dialysis [43]. In chronic kidney disease (CKD), a progressive condition prevalent in adults due to diabetes, hypertension, and glomerulonephritis, MSC therapy has been studied for its potential to slow disease progression. Adult trials have reported that MSCs, often delivered systemically, attenuate fibrosis, modulate immune responses, and stabilize glomerular filtration rates (GFR), with early‐phase studies indicating tolerability and modest efficacy [24].

The NEPHSTROM trial results published in 2023 represent a landmark advancement in MSC therapy for kidney disease. Perico et al. [44] demonstrated in a randomized clinical trial (NCT02585622) that MSC therapy notably reduced progression of eGFR deterioration over an 18‐month period compared to placebo, establishing a crucial precedent for pediatric applications. This breakthrough suggests that systematic MSC infusion may positively impact disease progression in younger patients with developing organ systems.

Updated 2023 evidence confirms that MSCs possess reparative and protective effects on kidney injury, functioning through secreting trophic factors and delivering extracellular vesicles with anti‐apoptotic, antioxidant, anti‐inflammatory, anti‐fibrotic, and immunomodulatory activities [45]. The therapeutic potential extends to viral‐induced AKI, where MSCs and their exosomes show promise as cell‐free methods for treating pediatric kidney injury through regenerative and anti‐inflammatory processes [46].

Pediatric‐specific advances reveal that recent findings from 2024 demonstrate that extracellular vesicles derived from renal autologous cells (RACev) effectively regulate post‐AKI fibrosis, inflammation, and hypoxia while promoting enhanced angiogenesis. Salybekov et al. [47] showed that transplanted RACev preferentially accumulate in ischemia‐injured proximal tubular cells, which are particularly vulnerable in pediatric patients. The REACT trials utilize allometric scaling based on kidney weight derived from 3D volumetric imaging, offering a more individualized approach with doses of 3 × 10⁶ cells/g of estimated kidney weight [48].

Beyond AKI and CKD, MSC therapy in adults has been applied to glomerular disorders, such as lupus nephritis and focal segmental glomerulosclerosis (FSGS), which are more common in adult populations due to autoimmune or secondary etiologies. Clinical studies have shown that MSCs reduce proteinuria and disease activity in lupus nephritis by suppressing T‐cell and B‐cell responses, with some patients achieving remission [49, 50]. In transplantation‐related kidney disease, such as delayed graft function (DGF) or chronic allograft nephropathy, MSCs have been used in adults to mitigate ischemia‐reperfusion injury and reduce rejection rates, leveraging their immunomodulatory properties [51]. Additionally, MSC therapy has been explored in related adult diseases outside nephrology that share pathophysiological features with kidney disease, such as diabetic nephropathy and liver cirrhosis. In diabetic nephropathy, MSCs have been shown to reduce hyperglycemia‐induced renal damage and fibrosis in adults [52], while in liver cirrhosis, they decrease hepatic fibrosis and inflammation, suggesting a broader regenerative potential [53].

Several critical differences distinguish MSC therapy's use in pediatric kidney diseases from adult applications, reflecting variations in disease etiology, patient physiology, and therapeutic considerations.

Firstly, the etiology of kidney diseases differs significantly between adults and children. In adults, AKI and CKD are predominantly driven by acquired conditions like diabetes, hypertension, and ischemic insults, whereas pediatric kidney diseases often stem from congenital anomalies (e.g., CAKUT) or genetic disorders (e.g., nephrotic syndrome due to NPHS1 mutations) [1]. This etiological divergence influences MSC therapy's goals, in adults, the focus is often on reversing or halting damage from secondary insults, while in children, it may involve supporting renal development and correcting structural defects [54].

Secondly, physiological differences between adults and children impact MSC therapy's application. Pediatric patients have smaller, developing kidneys with higher regenerative potential but greater vulnerability to procedural risks [24, 54]. In contrast, adult trials often employ intra‐arterial delivery to overcome pulmonary trapping and target larger, more fibrotic kidneys [24], a method less feasible in children due to smaller vasculature and increased procedural risk [39]. Furthermore, children's immune systems are less mature, potentially enhancing MSC engraftment due to reduced rejection but also raising concerns about long‐term immunogenicity.

Thirdly, disease progression and therapeutic endpoints differ. In adult CKD, the aim is often to delay progression to end‐stage renal disease (ESRD) in a fibrotic, aged kidney, whereas in pediatric CKD, preserving growth and development is paramount. For glomerular disorders, adults may require MSC therapy to manage chronic autoimmune activity (e.g., lupus nephritis), while pediatric cases, such as steroid‐resistant nephrotic syndrome, focus on stabilizing podocyte function early in life [55]. CAKUT, a uniquely pediatric condition, has no direct adult equivalent, and MSC therapy here aims to enhance nephrogenesis and prevent CKD onset, unlike adult applications focused on damage mitigation.

Finally, safety and ethical considerations differ significantly. Adult trials, such as those for AKI or transplantation, have established a broader safety profile for MSC doses and delivery methods [14], while pediatric applications remain in early‐phase trials with limited long‐term data. The ethical threshold for invasive procedures or experimental therapies is higher in children, necessitating non‐invasive options like intravenous injection over more invasive adult approaches like direct renal infusion. Early clinical trials demonstrate that MSC therapies yield significant functional improvements (e.g., creatinine reduction in AKI), warranting larger pediatric studies.

## 8 Conflicting Results and Limitations of Current Clinical Trials

Significant variability in MSC efficacy is evident across both preclinical and clinical studies, stemming from differences in study design, MSC sources, delivery methods, and disease‐specific factors. This heterogeneity complicates the interpretation of results and translation to clinical practice.

### 8.1 Preclinical Evidence

In preclinical models of acute kidney injury (AKI), bone marrow‐derived MSCs (BM‐MSCs) have consistently reduced serum creatinine and inflammatory markers (TNF‐α, IL‐6) by 30%–50% within 72 h post‐intervention [31]. However, source‐dependent variability is apparent; umbilical cord‐derived MSCs (UC‐MSCs) in cisplatin‐induced AKI rat models demonstrated significant improvements in glomerular filtration rate (GFR) and reduced apoptosis [1], while adipose‐derived MSCs (AD‐MSCs) showed less pronounced effects, with only approximately 20% reduction in creatinine and inconsistent fibrosis outcomes [54]. This discrepancy likely reflects intrinsic differences in MSC potency, as UC‐MSCs typically exhibit higher proliferation rates and immunomodulatory capacity compared to AD‐MSCs [56].

In chronic kidney disease (CKD) models, such as the 5/6 nephrectomy rat, AD‐MSCs reduced proteinuria by 40% and attenuated fibrosis [34], yet other investigations using BM‐MSCs failed to demonstrate significant GFR improvement. These contradictory findings suggest that disease stage or MSC senescence may be critical influencing factors [27]. Similarly, for glomerular disorders and congenital anomalies of the kidney and urinary tract (CAKUT), preclinical outcomes vary considerably; UC‐MSCs effectively reduced proteinuria in anti‐Thy1 glomerulonephritis [41], while CAKUT models (e.g., unilateral ureteral obstruction) showed only modest nephrogenesis enhancement with BM‐MSCs [40], potentially due to differences in underlying pathophysiology or intervention timing.

### 8.2 Clinical Study Heterogeneity

In clinical studies, result variability is even more pronounced, reflecting the heterogeneity of patient populations and trial designs. In pediatric AKI, an early‐phase trial demonstrated that allogeneic BM‐MSCs (1 × 10^6 cells/kg, intravenous) reduced creatinine by 25% and decreased dialysis requirements in children with sepsis‐ or chemotherapy‐induced injury [54]. Conversely, a parallel study in adults with post‐cardiac surgery AKI found no significant renal function improvement with similar BM‐MSC dosing, highlighting potential age‐ or etiology‐related differences in therapeutic response [43].

For CKD, UC‐MSCs stabilized estimated GFR (eGFR) and reduced albuminuria by approximately 30% in pediatric patients [39], yet a phase I/II trial in adults with diabetic nephropathy reported only transient eGFR improvements that diminished after 6 months [57]. In glomerular disorders, autologous AD‐MSCs reduced proteinuria by 40% in pediatric steroid‐resistant nephrotic syndrome (SRNS) [39], while a study in adults with lupus nephritis showed variable responses, with 30% of patients experiencing no benefit, possibly due to immune‐mediated resistance mechanisms [58].

Limited data on CAKUT suggest BM‐MSCs may improve renal cortical thickness [56], but small sample sizes (*n* = 6) and lack of controls significantly limit generalizability. These discrepancies underscore variability driven by multiple factors: MSC source (allogeneic vs. autologous), patient demographics (age, disease severity), and underlying pathophysiology (inflammatory vs. structural abnormalities).

### 8.3 Methodological Limitations

Several methodological limitations further complicate interpretation of MSC efficacy. First, small sample sizes in most studies restrict statistical power and generalizability. Second, the lack of standardized protocols for MSC preparation, characterization, dosing, and delivery routes hinders reproducibility. Current trials utilize various MSC sources (BM, UC, AD) at doses ranging from 1 to 2 × 10^6 cells/kg, delivered via different routes (intravenous, intra‐arterial, or local infusion) (Table 1), yet no consensus exists regarding optimal therapeutic regimens.

Third, relatively short follow‐up periods—typically 3–12 months [24, 39]—preclude comprehensive assessment of long‐term safety concerns (e.g., tumorigenicity) or durability of therapeutic benefits. Fourth, most published studies are early‐phase (I/II) trials focused primarily on safety rather than efficacy, with few randomized controlled trials (RCTs) specifically addressing pediatric kidney disorders [23]. This contrasts with adult studies, where RCTs have occasionally yielded null results [47], suggesting a critical need for more robust pediatric trial designs.

Finally, significant patient heterogeneity—spanning diverse etiologies within AKI (sepsis vs. chemotherapy), CKD (CAKUT vs. glomerulonephritis), and glomerular disorders (SRNS vs. lupus nephritis)—confounds outcome interpretation, as disease‐specific responses to MSC therapy vary considerably [55]. These limitations collectively highlight the preliminary nature of current evidence, necessitating larger, standardized, and longer‐term studies to definitively establish the efficacy of MSC therapy across pediatric kidney disorders.

## 9 Challenges and Future Directions

While MSC therapy holds great promise for treating pediatric kidney disorders, several key challenges must be addressed to advance this field.

### 9.1 Optimization of Cell Sources and Dosing

Determining the most effective source of MSCs and dosing regimens for various pediatric kidney disorders is crucial. Comparative studies are required to identify the optimal cell types and dosages for specific conditions, considering factors such as a child's body weight, kidney size, and disease severity. Both autologous and allogeneic approaches present distinct advantages and limitations that must be carefully evaluated for pediatric patients.

### 9.2 Standardization of Delivery Methods

The delivery methods for MSCs, including intravenous, intra‐arterial, and direct organ injection, need to be standardized to maximize therapeutic efficacy and minimize potential risks. While intravenous delivery offers simplicity and minimal invasiveness—particularly important for pediatric patients—it faces limitations from pulmonary trapping. Establishing standardized, age‐appropriate delivery protocols can lead to improved treatment outcomes while balancing efficacy with safety considerations unique to developing kidneys.

### 9.3 Long‐Term Safety and Efficacy Assessment

Comprehensive longitudinal studies are crucial to ensure MSC therapy safety for pediatric patients. Investigating potential risks such as tumorigenicity, immunogenicity, and unintended differentiation over extended periods is essential. Particular attention should focus on how MSC interventions might influence kidney development and function throughout childhood and into adulthood, providing valuable insights into the long‐term effects of this therapy.

### 9.4 Mechanistic Understanding

Gaining deeper insights into the mechanisms underlying MSC‐mediated renal repair and regeneration is vital for refining therapeutic approaches. Current knowledge gaps include understanding the relative contributions of paracrine signaling versus direct cellular integration and characterizing MSC interactions with the developing kidney microenvironment. Research into these specific mechanisms will aid in developing more targeted and effective treatments.

### 9.5 Regulatory and Ethical Frameworks

Addressing regulatory and ethical considerations related to MSC therapy is vital for ensuring safe and equitable access. This includes issues concerning cell sourcing, manufacturing processes, and obtaining informed consent from parents/guardians. International collaboration on regulatory frameworks would facilitate clinical translation while ensuring rigorous safety standards for this vulnerable population.

### 9.6 Specific Research Priorities for Advancing MSC Therapy

To translate MSC therapy into routine pediatric nephrology practice, specific research priorities must be pursued. First, identifying biomarkers for treatment response is essential to monitor efficacy and personalize therapy. Potential candidates include circulating IL‐10 and TGF‐β, reflecting MSC immunomodulation [18], and urinary KIM‐1 and NGAL, tracking repair in AKI and CKD [59], alongside EGF for regeneration in CAKUT [60]. Validating a comprehensive biomarker panel could address outcome variability [24, 38, 54] and guide appropriate dosing strategies.

Second, MSC‐engineered extracellular vesicles (EVs) offer a promising cell‐free alternative, leveraging paracrine effects while potentially reducing safety concerns [17]. Future studies should optimize EV isolation techniques, engineer specific therapeutic cargos like miR‐29a for glomerular repair [61], and test efficacy in pediatric‐focused trials. EVs may enhance delivery standardization [38] and overcome challenges associated with whole‐cell therapies.

Third, innovative approaches to enhance MSC therapeutic potential warrant investigation. MSC preconditioning methods (e.g., hypoxia) or genetic modification (e.g., HGF overexpression) could significantly boost efficacy, with preclinical evidence already demonstrating enhanced angiogenesis and fibrosis reduction [27, 34]. Establishing pediatric‐specific safety profiles for these enhanced cellular products remains essential before clinical implementation.

Finally, developing pediatric‐specific experimental models and trial frameworks will better align MSC applications with childhood kidney disease pathophysiology [56]. Models like neonatal ischemia‐reperfusion injury and the evaluation of combination therapies [34] with conventional treatments will provide more relevant insights for pediatric applications.

By systematically addressing these challenges and research priorities—from biomarkers and EVs to enhanced MSCs and tailored models—the field can advance toward more personalized, effective, and accessible MSC therapies for children with kidney disorders, potentially transforming treatment paradigms and improving long‐term outcomes.

## 10 Conclusion

Mesenchymal stem cell (MSC) therapy presents a promising frontier in managing pediatric kidney disorders, offering novel approaches beyond conventional treatments through multiple mechanisms: immunomodulation, tissue regeneration, anti‐fibrotic effects, and paracrine signaling. The cumulative evidence from both preclinical and early clinical studies demonstrates MSC potential in conditions ranging from acute kidney injury to chronic kidney disease, glomerular disorders, and congenital anomalies.

Despite encouraging preliminary results, several challenges remain before widespread clinical implementation. The optimal source selection—whether bone marrow, umbilical cord, or adipose tissue—significantly impacts therapeutic outcomes. Delivery methods greatly influence MSC biodistribution and efficacy, with systemic administration offering practicality but limited renal targeting compared to direct approaches. Pediatric applications require distinct considerations compared to adult populations, particularly regarding disease etiology (predominantly congenital/genetic vs. acquired), organ size and development, therapeutic goals, and heightened safety thresholds.

Current limitations include methodological heterogeneity across studies, small sample sizes, varying protocols, and insufficient long‐term safety data. Moving forward, research priorities should focus on identifying reliable biomarkers for treatment response, standardizing protocols, developing pediatric‐specific experimental models, optimizing delivery approaches, and establishing regulatory frameworks that address the unique needs of children with kidney disorders.

As this field progresses, collaboration between nephrologists, stem cell biologists, and regulatory bodies will be essential to translate promising research into effective, safe clinical therapies that could fundamentally transform outcomes for children with kidney diseases.

## Author Contributions

**Mahboube Bahroudi:** conceptualization, methodology, and writing – original draft. **Mastaneh Moghtaderi:** conceptualization, methodology, writing – review and editing, and supervision.

## Conflicts of Interest

The authors declare no conflicts of interest.

## Transparency Statement

The corrresponding author, Mastaneh Moghtaderi, affirms that this manuscript is an honest, accurate, and transparent account of the study being reported; that no important aspects of the study have been omitted; and that any discrepancies from the study as planned (and, if relevant, registered) have been explained.

## Data Availability

Data sharing not applicable to this article as no data sets were generated or analyzed during the current study.
